# Stereotactic Body Radiotherapy for Clinically Localized Prostate Cancer: Toxicity and Biochemical Disease-Free Outcomes from a Multi-Institutional Patient Registry

**DOI:** 10.7759/cureus.395

**Published:** 2015-12-04

**Authors:** Joanne Davis, Sanjeev Sharma, Richard Shumway, David Perry, Sean Bydder, C. Kelley Simpson, David D'Ambrosio

**Affiliations:** 1 Clinical Programs, The Radiosurgery Society; 2 Department of Radiation Oncology, St. Mary's Medical Center; 3 Department of Radiation Oncology, Saint Francis Hospital and Medical Center; 4 Radiation Oncology, Medstar Franklin Square Medical Center, Baltimore, MD; 5 Department of Radiation Oncology, Sir Charles Gairdner Hospital, Perth, Australia; 6 Radiation Oncology, Colorado CyberKnife, Lafayette, CO; 7 Radiation Oncology, Community Medical Center-Barnabas Health; 8 NJ Cyberknife, Community Medical Center-Barnabas Health

**Keywords:** prostate cancer, stereotactic body radiotherapy, sbrt, psa, prostate-specific antigen, genitourinary malignancies, registry, aging, disease-free survival

## Abstract

Objectives: To report on initial patient characteristics, treatment practices, toxicity, and early biochemical disease-free survival (bDFS) of localized prostate cancer treated with stereotactic body radiotherapy (SBRT) and enrolled in the RSSearch^®^ Patient Registry.

Methods: A retrospective analysis was conducted on patients with clinically localized prostate cancer enrolled in RSSearch^®^ from June 2006 - January 2015. Patients were classified as low-risk (PSA ≤ 10 ng/ml, T1c-T2a, Gleason score ≤ 6), intermediate-risk (PSA 10.1 - 20 ng/ml, T2b-T2c, or Gleason 7), or high-risk (PSA > 20 ng/ml, T3 or Gleason ≥ 8). Toxicity was reported using Common Toxicity Criteria for Adverse Events, version 3. Biochemical failure was assessed using the Phoenix definition (nadir + 2 ng/ml). The Kaplan-Meier analysis was used to calculate bDFS and association of patient and tumor characteristics with the use of SBRT.

Results: Four hundred thirty-seven patients (189 low, 215 intermediate, and 33 high-risk) at a median of 69 years (range: 48-88) received SBRT at 17 centers. Seventy-eight percent of patients received 36.25 Gy/5 fractions, 13% received 37 Gy/5 fractions, 6% received 35 Gy/5 fractions, 3% received 38 Gy/4 fractions, and 5% received a boost dose of 19.5-29 Gy following external beam radiation therapy. Median follow-up was 20 months (range: 1–64 months). Genitourinary (GU) and gastrointestinal (GI) toxicities were minimal, with no acute or late Grade 3+ GU or GI toxicity. Late Grade 1 and 2 urinary frequency was 25% and 8%. Late Grade 1 and 2 proctitis was 3% and 2%. Median PSA decreased from 5.8 ng/ml (range: 0.3-43) to 0.88, 0.4, and 0.3 ng/ml at one, two, and three years. Two-year bDFS for all patients was 96.1%. Two-year bDFS was 99.0%, 94.5%, and 89.8% for low, intermediate, and high-risk patients (p < 0.0001). Two-year bDFS was 99.2%, 93.2%, and 90.4% for Gleason ≤ 6, Gleason 7, and Gleason ≥ 8 (p < 0.0001). Two-year bDFS was 96.4%, 97.2%, and 62.5% for PSA ≤ 10 ng/ml, PSA 10.1 - 20 ng/ml, and PSA > 20 ng/ml (p < 0.0001). Clinical T Stage was not significantly associated with bDFS.

Conclusions: Early disease outcomes of SBRT for the treatment of clinically localized prostate cancer from a multicenter patient registry compare favorably with reports from single institutions. Acute and late GU and GI toxicities were minimal, and PSA response to SBRT was highly encouraging. Continued accrual and follow-up will be necessary to confirm long-term results.

## Introduction

Prostate cancer remains the most common cancer diagnosed in men with an estimated 220,800 newly diagnosed cases and 27,540 estimated deaths in the US in 2015 [1]. The majority of prostate cancer patients are diagnosed with clinically localized disease for which the five-year survival rate is near 100%. For patients with early-stage prostate cancer and a life expectancy greater than 10 years, treatment management options may include active surveillance, radical prostatectomy, external beam radiation therapy, and brachytherapy [2]. Studies have shown similar survival and bDFS rates for early-stage prostate cancer patients treated with these treatment methods [3-10]. Treatment, however, often impacts the quality of life due to side-effects and treatment-related toxicities, such as urinary and bowel complications as well as erectile dysfunction [11].

Over the past decade, radiation techniques involving intensity-modulated radiation therapy (IMRT) and imaged-guided radiation therapy (IGRT) have evolved to allow safe administration of higher doses of radiation. Results of randomized trials have suggested that dose escalation is associated with improved biochemical outcomes, with doses of 75.6 Gy to 79.2 Gy recommended for patients with low-risk prostate cancer, and up to 81 Gy for patients with intermediate to high-risk disease [5-6, 12-13]. Disadvantages of external beam radiation, however, include long treatment courses over eight to nine weeks, risks of  bladder and/or bowel symptoms during and after treatment, and the risk of erectile dysfunction [13]. 

Stereotactic body radiotherapy (SBRT) is a technique that delivers highly conformal, high-dose radiation in a few treatment fractions (hypofractionation), typically 4 to 5 fractions for prostate cancer. Tissues with a low α/β ratio are more sensitive to large doses of radiation per fraction [14-15]. The α/β ratio for prostate cancer is thought to be low (range: 1.5 - 3) while surrounding normal tissues have a higher α/β ratio (range: 3 – 8) [16-18]. These reports suggest there is a potential therapeutic gain with hypofractionation for the treatment of prostate cancer, where large doses per fraction of radiation are expected to result in improved tumor control with limited toxicity. Studies describing the treatment of low- and intermediate-risk prostate cancer with SBRT have been promising, with five-year bDFS ranging from 84 - 98% [19-25]. Katz, et al. reported seven-year bDFS for low, intermediate, and high-risk disease of 95.6%, 89.3%, and 68.5%, respectively, with low genitourinary and gastrointestinal toxicities [26]. In 2015, the National Comprehensive Cancer Network (NCCN) updated the NCCN Guidelines for Prostate Cancer to include SBRT regimens (6.5 Gy per fraction or greater) as “an emerging treatment modality and can be considered as an alternative to conventionally fractionated regimens at clinics with appropriate technology, physics, and clinical experience” [2].

The use for SBRT for the treatment of clinically localized prostate cancer is growing in the community setting [27-28]. As the use of SBRT for the treatment of clinically localized prostate cancer continues to expand, it will be important for treatment centers to capture and report treatment management practices and clinical outcomes to ensure continued efficacy and compliance to standardized protocols and published treatment guidelines. The purpose of this study was to report the initial findings of SBRT treatment management practices, toxicity, and early clinical outcomes from patients with clinically localized prostate cancer treated with SBRT and enrolled in a multicenter patient registry, which includes both academic and community-based radiation therapy practices. 

## Materials and methods

A retrospective analysis of patients with clinically localized prostate cancer treated with SBRT and enrolled in the RSSearch^®^ Patient Registry (ClinicalTrials.gov Identifier: NCT01885299) between June 2006 and January 1, 2015 was performed. The RSSearch^®^ Patient Registry is managed by the Radiosurgery Society^®^, a non-profit professional medical society. A description of the methodology, database design, and initial patient and treatment characteristics of patients enrolled in RSSearch^®^ has been previously reported [29]. The database is housed by an independent third-party, Advertek^SM^ Inc. (Louisville, KY) and meets all requirements to comply with the Health Insurance Portability and Accountability Act (HIPAA) and Safe Harbor Policy to maintain system security, transmission of data, and patient confidentiality. All centers treating patients with SBRT clinically are offered and encouraged to participate in RSSearch^®^. Participation is voluntary and no compensation is provided either to patients or participating centers. Each principal investigator is provided a copy of the RSSearch^®^ registry protocol, case report forms, sample patient informed consent, and web-based training for data entry and database navigation. Local Institutional Review Board/Ethics Committee (IRB/EC) approval is required at all participating centers. Informed consent was obtained from all patients, as required by individual IRB/ECs, prior to the patient’s data entered into the RSSearch^®^ registry. The Institutional Review Board/Ethics Committee of Community Medical Center, Barnabas Health approved this study (#11-018). For this analysis, RSSearch^®^ was screened for patients with clinically localized low-risk (PSA ≤ 10 ng/ml, Gleason ≤ 6, Clinical T Stage T1c-2a, N0, M0), intermediate-risk (PSA 10.1 -20 ng/ml, Gleason 7 or Clinical T Stage T2b-T2c, N0M0) and high-risk (PSA > 20 ng/ml, Gleason ≥ 8 or Clinical T Stage T3-4) disease with PSA follow-up data. We did not classify patients as “very low risk” or “very high risk” as per NCCN 2015 criteria because the PSA density and number of involved biopsy cores were not available in RSSearch. Patients with positive lymph nodes or evidence of metastatic disease were excluded from the analysis. Only patients with complete SBRT treatment information were included in the analysis. One hundred and twenty-three patients were excluded due to missing SBRT treatment information.

Because this is a registry, there were no pre-defined treatment planning criteria, and treatment planning was done per institutional guidelines. All patients were simulated and treated in the supine position. Planning computed tomography (CT) scans were obtained above and below the treatment region. One mm slice thickness reconstructions in the axial plane were transferred to the treatment planning station. Magnetic resonance imaging (MRI) scans were routinely used for image fusion to aid target volume delineation. Target volumes were delineated by the treating physician using all available imaging studies, typically including at least CT and MRI scanning. The gross tumor volume (GTV) was generally used as the clinical target volume (CTV), and a margin of 1-5 mm was used to delineate the planning target volume (PTV). All patients were treated according to the respective institutional guidelines and treated with either fiducial-based image-guided CyberKnife robotic radiosurgery or linac-based systems. Treatment characteristics were captured on the RSSearch® case report forms. 

Patient follow-up was performed per institutional guidelines. All participating centers reported follow-up clinical evaluations and serum PSA levels. Patients were excluded from the analysis if they did not have a minimum of one PSA follow-up value post-SBRT treatment. Biochemical disease-free survival (bDFS) was evaluated using the Radiation Therapy Oncology Group Phoenix definition of a PSA rise of 2 ng/ml or more above the nadir PSA. PSA bounce was defined as PSA increase of ≥ 0.4 ng/ml between any two consecutive measurements followed by a subsequent decline to or below the previous nadir. Descriptive statistics were performed for patient and treatment characteristics. Analyses of bDFS were calculated using the Kaplan-Meier method. The log-rank test was used to compare bDFS between subgroups. Values of p < 0.05 were considered statistically significant. Statistical calculations were conducted using GraphPad Prism (La Jolla, CA) and STATA (StatCorp LP, TX).

## Results

### Patient characteristics

A total of 437 patients treated with SBRT at 16 community-based and academic centers in the US and one academic center in Australia were included in the analysis. The median follow-up was 20 months (range: 1 - 64 months). Patient characteristics are shown in Table 1. The median age was 69 years (range: 48 - 88 years), median prostate volume was 52 cc (range: 10 - 180 cc), and median baseline PSA was 5.8 ng/ml (0.3 - 43 ng/ml). Clinical stage was T1a - T1c in 341 patients (79%), T2a in 70 patients (16%), T2b in 18 patients (4%), T2c in five patients (1%), and T3 in three patients (1%). Gleason score was ≤ 6 in 222 (51%) patients, Gleason score 3+4=7 in 154 (44%) patients, Gleason 4+3=7 in 38 (9%) patients, and Gleason score ≥ 8 in 23 (5%) patients. One-hundred and eighty-nine patients (43%) had low-risk disease, 215 (49%) had intermediate-risk disease, and 33 patients (8%) had high-risk disease. The majority of patients were Caucasian (86%), 10% were African-American, 2% Asian, and 2% Hispanic. Men were most commonly referred to SBRT by their primary urologist (74%), 15% were self-referrals, and 11% were referred to SBRT by either primary care, medical oncology, or other physician specialists. Eleven percent of patients received hormone therapy, including 10 low-risk, 23 intermediate-risk, and 15 high-risk patients. Patients were placed on hormones at the urologist's discretion prior to being referred to radiation oncology for SBRT.

**Table 1:** Baseline patient and tumor characteristics and SBRT treatment

Variable (n=437)	n (%)
Median age (range), years	69 (48-88)
Median Karnofsky Performance Score (range)	100% (60-100%)
Ethnicity:	
Caucasian	373 (85%)
African American	45 (10%)
Asian/Pacific-Asian	7 (2%)
Other	5 (1%)
Not reported	7 (2%)
Median prostate volume (range), cc	52 (10-180)
Median initial PSA (range), ng/ml:	5.8 (0.3-43)
≤ 10	373 (86%)
10-20	58 (13%)
> 20	6 (1%)
Gleason score:	
≤ 6	222 (51%)
3+4	154 (36%)
4+3	38 (9%)
≥ 8	23 (5%)
Clinical T stage:	
T1a-1c	341 (79%)
T2a	70 (16%)
T2b	18 (4%)
T2c	5 (1%)
T3	3 (1%)
Risk group:	
Low	189 (43%)
Intermediate	215 (49%)
High	33 (8%)
Median SBRT dose (range), Gy	36.25 (19-38)
Dose/fractionation schema:	
19.5 – 29 Gy/2-3 fractions	23 (5%)
35 Gy/5 fractions	24 (6%)
36.25 Gy/ 5 fractions	328 (76%)
37 Gy/5 fractions	58 (13%)
38 Gy/ 4 fractions	4 (1%)
Median maximum point dose (range), Gy	46.47 (24-76)

### SBRT treatment

The majority of patients were treated with SBRT as monotherapy with doses ranging from 35 - 38 Gy delivered in 4-5 fractions. Three hundred and twenty-eight patients (76%) received 36.25 Gy in 5 fractions, 13% of patients received 37 Gy in 5 fractions, 6% received 35 Gy in 5 fractions, and 1% received 38 Gy in 4 fractions (Table 1). A small percent of patients (5%) received 19.5 - 29 Gy delivered in 2-3 fractions as a “boost” following external beam radiotherapy. All patients that received SBRT as a boost were intermediate- or high-risk.

### Toxicity

The incidence of acute and late GU and GI toxicity following SBRT was low. There were no Grade 3 or higher acute or late GU or GI toxicities reported. Table 2 shows the incidence of acute GU and GI toxicity according to CTCAE v3. The most common acute GU toxicity was urinary frequency, with acute Grade 1 and Grade 2 urinary frequency reported in 19% and 2% of patients, respectively. Acute Grade 1 and Grade 2 urinary retention was reported in 3% and 1% of patients, respectively. The most commonly reported acute GI toxicity was diarrhea. Acute Grade 1 and 2 diarrhea was reported in 4% and 1% of patients, respectively. Late GU and GI toxicities were minimal (Table 3). Late Grade 1 and 2 urinary frequency was reported in 25% and 8% of patients, respectively. Late Grade 1 and 2 proctitis was reported in 3% and 2% of patients, respectively. Erectile dysfunction was not assessed in this study. Erectile function prior to SBRT treatment was not reported in RSSearch® and changes in erectile function or potency after SBRT could not be evaluated. Late Grade 1 pain was reported in 4% of patients and one patient had a late Grade 3 pain toxicity. There were no other late Grade 3 or higher toxicities.

**Table 2:** Acute toxicity following SBRT using CTCAEv3 grading system

Acute Toxicity			
Symptom	Grade 1	Grade 2	Grade 3 – 5
Urinary frequency	19%	2%	0%
Urinary retention	3%	1%	0%
Cystitis	3%	1%	0%
Diarrhea	4%	1%	0%
Constipation	1%	0%	0%
Proctitis	1%	0%	0%
Fatigue	2%	0%	0%
Pain	3%	1%	0%

**Table 3:** Late toxicity following SBRT using CTCAEv3 grading system

Late Toxicity				
Symptom	Grade 1	Grade 2	Grade 3	Grade 4-5
Urinary frequency	25%	8%	0%	0%
Urinary retention	4%	2%	0%	0%
Cystitis	5%	2%	0%	0%
Diarrhea	4%	0%	0%	0%
Constipation	3%	0%	0%	0%
Proctitis	3%	2%	0%	0%
Fatigue	3%	0%	0%	0%
Pain	4%	0.2%	0.2%	0%

### PSA trend

**Figure 1:**
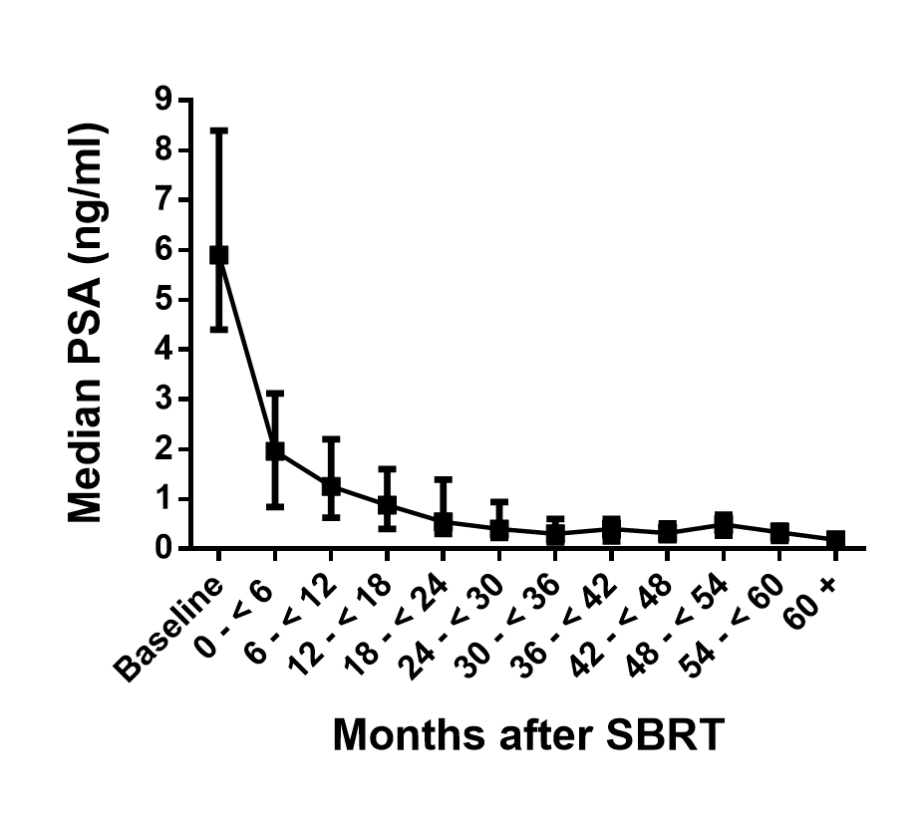
Median PSA (ng/ml) response at baseline and at indicated time points after SBRT treatment for all patients. Interquartiles are shown as error bars.

Median PSA declined from a baseline value of 5.9 ng/ml to 0.88 ng/ml at 12 months, representing an 85% decline in PSA over the first year (Figure 1). PSA continued to decrease over time with a median PSA value of 0.4 and 0.3 ng/ml at two and three years, respectively. There was no difference in PSA nadir when stratified by a low vs. intermediate risk group at one, two, or three years duration after SBRT (p = 0.89 by log-rank test).

We examined PSA bounce using the criteria of PSA increase of ≥ 0.4 ng/ml between any two consecutive measurements followed by a subsequent decline to or below the previous nadir described by McBride, et al. [23]. Thirty-five (6.6%) patients had a reported PSA bounce. The median time to PSA bounce was 15 months (range: 6 - 52 months) and the median PSA bounce value was 2.2 (range: 0.6 - 6.9 ng/ml). The median age of those who experienced a bounce was significantly younger (66 years vs. 70 years, p = 0.049) compared to those who did not experience a PSA bounce. Initial PSA (p=0.07) and risk group (p=0.59) did not predict for PSA bounce.

### Biochemical disease-free survival

**Figure 2:**
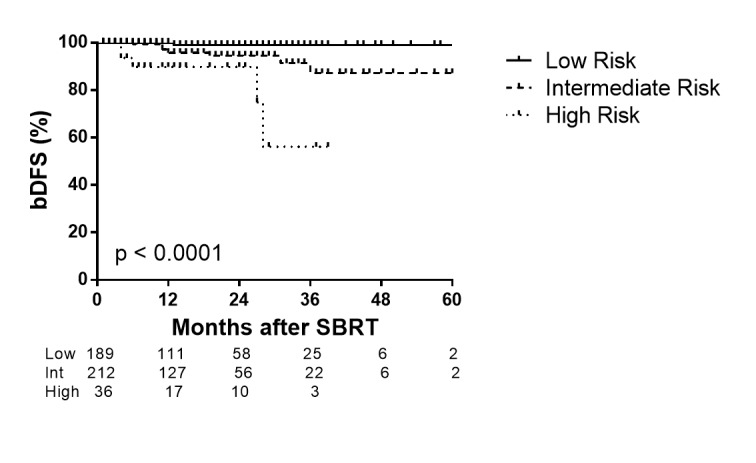
Rate of biochemical disease-free survival for low, intermediate, and high-risk patients after SBRT. Number of subjects for each risk group are shown below, p value < 0.0001 by log-rank test. Tick marks indicate censored patients.

The two-year bDFS for the entire cohort was 96.1%. When stratified by risk group, two-year bDFS for low, intermediate, and high-risk patients was 99.0%, 94.5%, and 89.8%, respectively (p < 0.0001 by log-rank test) (Figure 2). The three-year bDFS was 99.0% and 91.4% for low and intermediate-risk, respectively. Fifteen patients had biochemical failures using the Phoenix definition of biochemical failure (nadir + 2 ng/ml). One low-risk patient (T1c, Gleason 6, pre-treatment PSA 8.85 ng/ml) had a PSA increase from a nadir of 2.2 ng/ml at seven months to 6.8 ng/at 13 months, which met the criteria of nadir + 2 definition of biochemical failure. Eight intermediate-risk (4%) patients failed at a median of 18 months (range: 6 - 36 months), and six high-risk patients (6%) failed at a median of 17 months (range: 4 - 31 months). 

**Figure 3:**
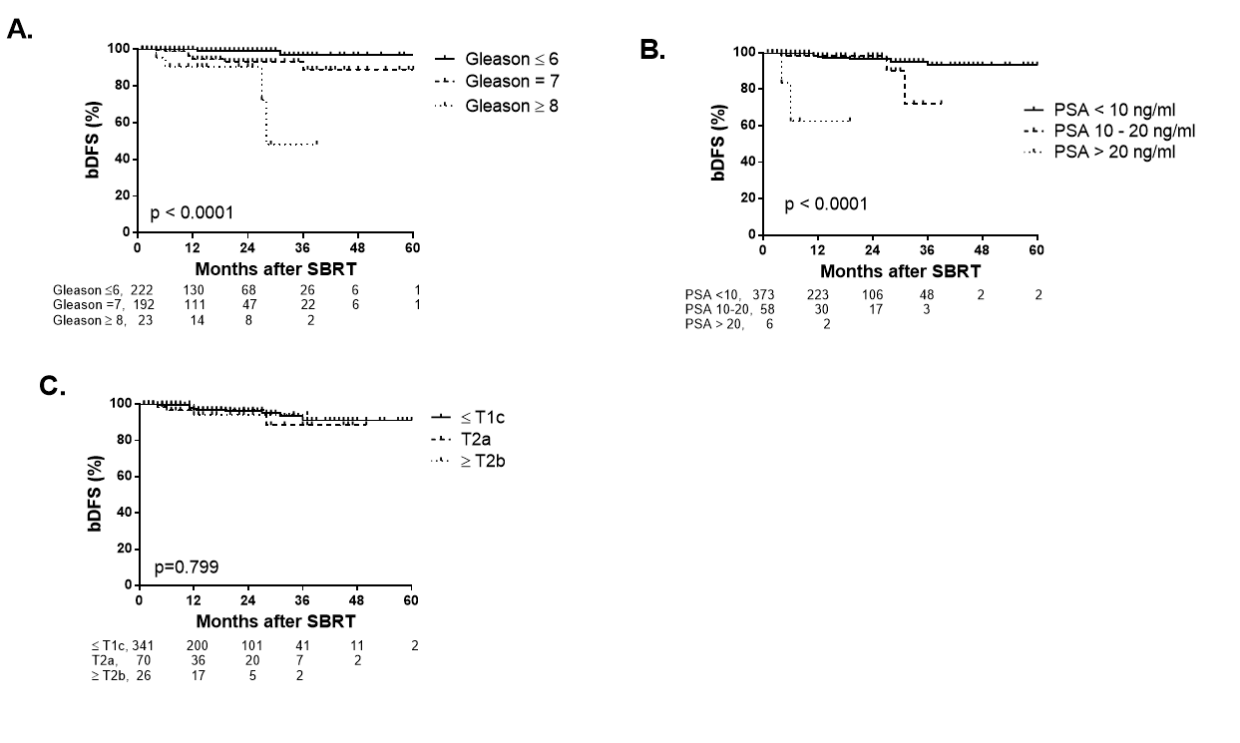
Rate of bDFS stratified by Gleason Score (A), pre-treatment PSA (B) and Clinical T Stage (C). Number of subjects are shown below. Tick marks indicate censored patients.

When patients were stratified by Gleason score (Gleason ≤ 6, Gleason 7, and Gleason ≥ 8), higher Gleason score was significantly associated with lower bDFS (p < 0.0001 by log-rank test) (Figure 3A). The two-year bDFS for Gleason ≤ 6, Gleason 7, and Gleason ≥ 8 was 99.2%, 93.2%, and 90.4%, respectively. Among patients in the intermediate risk group, there was not a significant difference for bDFS between patients with Gleason 3+4 and Gleason 4+3 (p=0.725 by log-rank test). When patients were stratified by initial PSA (PSA ≤ 10 ng/ml, PSA 10.1 - 20 ng/ml, and PSA > 20 ng/ml), patients with PSA > 20 ng/ml were significantly associated with lower bDFS (p < 0.0001 by log-rank test) (Figure 3B). Two-year bDFS rates for patients with PSA ≤ 10 ng/ml, PSA 10.1 – 20 ng/ml, and PSA > 20 ng/ml were 96.4%, 97.2%, and 62.5%, respectively. When stratified by Clinical T Stage, there was not a significant difference for bDFS between patients with Clinical Stage T1c or less compared to Stage T2a-2b or ≥ T2c (p = 0.799) (Figure 3C).

## Discussion

In the current study, we described the screening, treatment management practices, toxicity, and early bDFS in patients with clinically localized prostate cancer treated with SBRT and enrolled in the RSSearch^®^ Patient Registry. The majority of patients treated with SBRT had low (44%) and intermediate-risk (49%) disease. Eight percent of patients had high-risk disease and received SBRT as either monotherapy or as a boost in conjunction with conventionally fractionated EBRT. For patients that received SBRT monotherapy, the most common SBRT dose/fractionation regimen was 36.25 Gy delivered in 5 fractions. Other treatment regimens included 35 Gy in 5 fractions, 37 Gy in 5 fractions, and 38 Gy in 4 fractions. This is consistent with published reports, where the majority of SBRT data to date include low and intermediate-risk patients treated with 35 - 36.25 Gy delivered in 5 fractions [20-22, 30-31].   

In our current study, acute and late GU and GI toxicity were low. Minimal GU and GI toxicities have also been reported in other studies using SBRT doses of 35 - 36.25 Gy delivered in 5 fractions [19, 24, 30-34]. King, et al. reported urinary Grade 1, 2, and 3 toxicity in 23%, 5%, and 3% of patients, respectively, and Grade 1 and 2 rectal toxicity in 12.5% and 2% of patients, respectively [33]. Chen, et al. reported 2-year incidence of Grade 2 or greater GU toxicity of 31% and Grade 2 GI toxicity of 1% in patients treated with 35 – 36.25 Gy at Georgetown University [31]. There were no Grade 3 or greater GI toxicities and only one Grade 3 hematuria. In 2013, Katz, et al. published one of the most mature series to date, which included 305 low, intermediate, and high-risk patients treated with 35 - 36.25 Gy in 5 fractions [35]. In this study, there were no acute Grade 3 or greater urinary or rectal complications; however, patients that received the higher dose of 36.25 Gy had a greater incidence of late Grade 2 and 3 urinary toxicities and late Grade 2 rectal toxicity, suggesting that a higher SBRT dose was associated with a higher incidence of late urinary and rectal toxicity. In a Phase I/II dose escalation study, prostate cancer patients receiving 50 Gy in 5 fractions had higher rates of late Grade 2 and 3 GU and GI toxicity compared to patients that received lower doses [32]. An updated report of the Phase I/II study indicated that 6.6% of patients that received 50 Gy in 5 fractions developed high-grade rectal toxicity, with five patients requiring colostomy [36]. The late Grade 3 or greater rectal toxicity correlated with the rectal wall receiving 50 Gy > 3 cm. Bernetich, et al. showed improved freedom from biochemical failure in intermediate and high-risk patients treated with 37.5 Gy compared to 35-36.25 Gy; however, patients receiving 37.5 Gy experienced slightly higher Grade 3 GU toxicity [34]. In our study, we were not able to stratify by dose because the majority of patients were treated with 36.25 Gy in 5 fractions, and there were limited numbers of patients treated with higher doses, thus, limiting our ability to conduct analysis with adequate statistical power to assess correlations between dose and incidence of toxicity.  

We demonstrate that SBRT delivered in a community practice setting achieves favorable bDFS for low, intermediate, and high-risk patients with two-year bDFS rates of 99%, 94.5%, and 89.9%, respectively. We further analyzed correlations between pre-treatment factors and bDFS and showed that both a higher Gleason score and pre-treatment PSA were predictors for biochemical failure. These results are in line with other single institution and pooled reports of SBRT for prostate cancer [19-24, 30-34]. Madsen, et al. published one of the early reports describing SBRT (33.5 Gy in 5 fractions) for the treatment of clinically localized prostate cancer [19]. Forty-eight month biochemical freedom from relapse (nadir + 2 ng/ml definition) was 90%. In 2010, Freeman and King published their combined data, including 41 low-risk patients treated with 35 – 36.25 Gy in 5 fractions [20]. The five-year biochemical progression-free survival was 93%. In a pooled analysis, including 1,100 patients treated with a median of 36.25 Gy in 5 fractions, the five-year biochemical relapse-free survival for low, intermediate, and high-risk patients was 95%, 84%, and 81%, respectively [22]. When stratified by Gleason score, five-year relapse-free survival was 95%, 83%, and 78% for Gleason ≤ 6, Gleason 7, and Gleason ≥ 8, consistent with our results. 

A rapid rate of PSA decline and a low PSA nadir (≤ 0.5 ng/ml) have been shown to predict favorable clinical outcomes after radiation therapy [37-38]. Several reports have shown a rapid decline of PSA over the first year after SBRT with a continued decline over the next two to three years, consistent with our results [23, 30, 34, 39]. Anwar, et al. further demonstrated that SBRT produced a lower PSA nadir and greater rate of decline in PSA over the same period of time compared to conventionally fractionated EBRT [39]. In our study, we showed an 85% decline in median PSA over the first 12 months after SBRT with a median PSA of 0.88 ng/ml. PSA continued to decline through years two (median PSA 0.4 ng/ml) and three (median PSA 0.3 ng/ml). Although our follow-up data is relatively short, Zelefsky, et al. demonstrated that PSA nadir ≤ 1.5 ng/ml at two years was predictive for distant metastases and cause-specific mortality [40]; thus, our short-term results may be predictive of long-term outcomes. We will continue to follow-up patients in RSSearch® and will conduct future studies on PSA kinetics with longer follow-up.

It is important to note that this study has several limitations. First, this study is limited by its retrospective nature and relatively short follow-up. We did not report patient reported quality-of-life in this study as patient reported outcomes are currently not captured in RSSearch®. We did not report on erectile dysfunction or potency rates at this time, as potency prior to SBRT was not captured in RSSearch®. Complete dose volume histogram (DVH) information was not available for all patients, and correlations between toxicity and doses to organs at risk (bladder and rectum) could not be evaluated at this time. It is also important to note that a potential limitation of our study, as with all patient registries, is the potential for under-reporting. We cannot exclude the possibility that some toxicities may not have been recorded in the RSSearch® database and, thus, are not represented in this analysis. 

## Conclusions

Compared to conventional external beam radiation, SBRT for prostate cancer may have several potential advantages, including a therapeutic advantage based on radiobiological effects [14, 17], convenience, reduced treatment times, cost effectiveness [41], and patient preference. All of these factors make SBRT an attractive treatment option for clinically localized prostate cancer. As the use of SBRT to treat prostate cancer expands, it is crucial to continue to capture treatment management practices and report on clinical outcomes in both the academic and community-based practices. In this study, we demonstrate that SBRT can be delivered safely in community based-practices, with initial PSA outcomes appearing favorable. We will continue to maintain and enroll prostate cancer patients in RSSearch®, collect follow-up data with quality assurance measures, and plan future studies on long-term data.
